# Bridging skill gaps and creating future ready accounting and finance graduates: an exploratory study

**DOI:** 10.12688/f1000research.72880.1

**Published:** 2021-09-06

**Authors:** Saravanan Muthaiyah, Karen Phang, Sanjaya Sembakutti

**Affiliations:** 1 Faculty of Management, Multimedia University, Cyberjaya, Selangor, 63100, Malaysia; 2 CIMA SE Asia Sdn Bhd, AICPA, Bandar Utama, Selangor, 47800, Malaysia

**Keywords:** Digital transformation, Pedagogy, FinTech, Teaching and Learning.

## Abstract

**Background:** Changing trends in the use of technology have become an impelling force to be reckoned with for the accounting and finance profession. The curriculum offered in higher learning institutions must be quickly revamped so that students who complete a bachelor’s degree are digitally competent upon graduation. With US$55.3 billion invested in FinTech in 2019 alone and more than 72% of accounting jobs being automated, graduates must be trained on digital skills to be future proof. Accounting and finance graduates must be made competent in skills that are related to digital content such as blockchain technology, information assets and autonomous peer to peer systems, to name a few.

**Methods:** We used a three-phase approach: 1) careful mapping of digital topics taught within the course structure offered at these institutions; 2) review of current best practices and digital learning tools for digital inclusion which was ascertained from literature; and 3) 80 experts in a think tank group were interviewed on antecedents, awareness and problems in relation to digital inclusion within the curriculum to validate our research objective.

**Results: **Eleven key tools for inclusion in the curriculum were discussed with experts and then mapped to current curriculum offered at institutions. We discovered that less than 5% of these were being taught. In total, 78% of experts agreed that digital content is inevitable, 90% agreed that digital inclusion based on tools that were discussed will yield great benefits for students, and lastly 75% agreed that giving digital exposure to students must be standard practice.

**Conclusions: **The response from experts confirms that digital inclusion is imperative, but instructors themselves lacked the know-how of emerging technologies. Only the curriculum of institutions with approved bachelor’s programs were included in this research. In our future work we hope to include all institutions and professional bodies as well.

## Introduction

Fintech is a fusion of emerging technologies and finance to provide greater insights to the practice of accounting and finance. However, academic content in the curriculums offered in degree programs do not include these digital topics due to multiple factors, and this has caused education programs to have suffered severe criticism over recent years.

### Literature review

Moffitt listed 30 emerging technologies that will have an enormous impact on jobs and artificial intelligence (AI) was the top of that list. ^1^ Blockchain has been listed as the 4 ^th^ most prominent emerging technology that will impact jobs. ^2^ Blockchain platforms resolve the core trust issue by maintaining a shared distributed ledger. ^3, 4^ Smart contracts within a blockchain can enable the transfer of titles, physical goods and assets simultaneously. Given that a sharing economy will be embedded in every financial system by 2020, financial systems will operate like any other sharing economy.

This gives rise to cloud computing which would perhaps have the second highest impact on accounting jobs. ^5^ Artificial intelligence (AI) has also found its way into predictive models. ^6^ Audit tasks will also benefit from AI, as the use of artificial neural networks (ANN) for audit tasks thus increases the ability to track and uncover control flaws that humans can’t. ^4, 7^ There is a high demand for digital skills, however due to the lack of digital skill sets companies aren’t able to hire a skilled tech-savvy financial workforce. ^8^ Employers have demonstrated their disappointment as the digital gap widens among finance graduates. ^9^ In a study conducted on 200 accounting and finance firms, where 105 graduate students from different universities were evaluated for their digital know-how, results indicated that these graduate students largely lacked in analytical and other digital skills. Hiring personnel equipped with skills in finance-related software, data analytics, and modelling programs is becoming difficult because of the outdated curriculum, which focuses more on traditional and obsolete concepts. ^4, 10^

### Descriptive, prescriptive and predictive accounting

**Figure 1.**
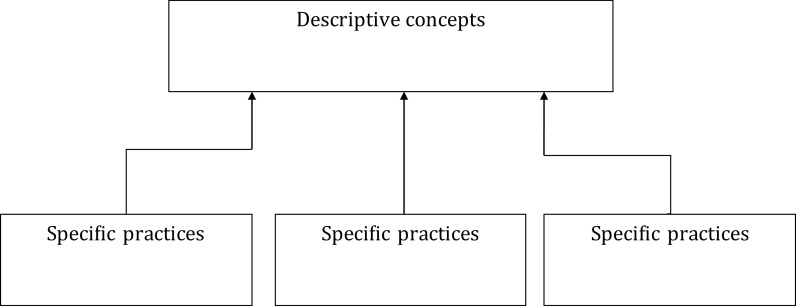
Descriptive approach to the bottom-up approach.

**Figure 2.**
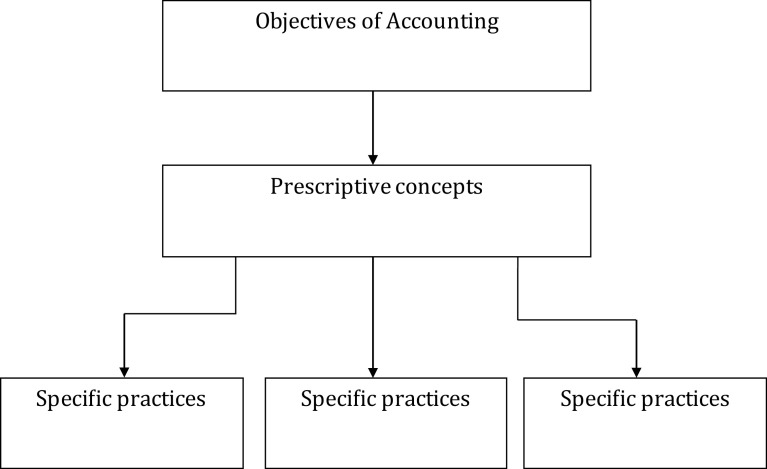
Prescriptive approach or top-down approach.

Today’s accounting curriculum is focused on descriptive accounting (see Figure 1). ^3^ Prescriptive accounting is rather limited, with the exception of managerial and cost accounting only (see Figure 2). For example, assets are still recorded and maintained at cost which is not aligned to fair value accounting principles.

**Figure 3.**
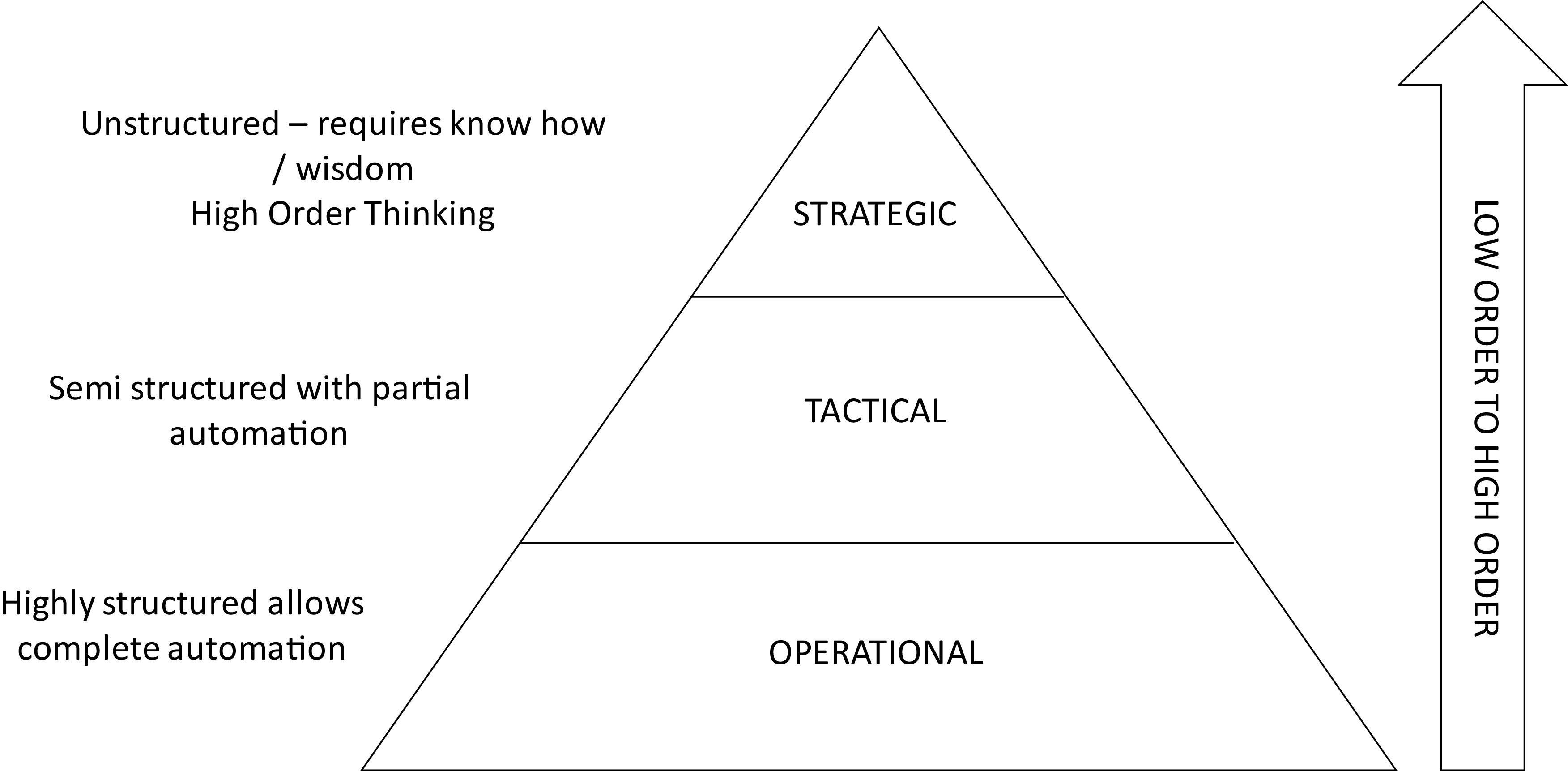
From descriptive to prescriptive roles.

At the operational level most activities can be fully automated. Activities at this level, such as journal entries, are mostly descriptive. The tactical layer requires some level of automation but will mostly be prescriptive (see Figure 3).

### Course mapping for digital inclusion

Based on the earlier discussion, we proceeded to attempt to understand how much of these essential digital topics (see Table 1) were embedded in the accounting curriculum, as these tools have been endorsed by the literature. Upon mapping to the existing curriculum of major accredited institutions we found very little or no evidence of these digital tools being embedded into the taught curriculum. Out of 11 teaching technologies or tools we could only partially map one item. Table 1 summarizes our findings.

**Table 1.** Classroom technologies for digital skills.

Items	Classroom technologies for future accountants	Mapped to existing curriculum
1	SAP (ERP- enterprise resource planning)	Yes - partially
2	IBM Cloud	Not traceable
3	Bloomberg	Not traceable
4	Microsoft Dynamics 365	Not traceable
5	XBRL (Extensible Business Reporting Language)	Not traceable
6	Idaciti	Not traceable
7	Aura and e-audit	Not traceable
8	DMS (document management system)	Not traceable
9	AIS (accounting information systems)	Not traceable
10	Cyber security and trusted computing	Not traceable
11	CEPS (cryptocurrencies and electronic payment systems)	Not traceable

### Study objectives and research questions

The objective of this section was to ascertain technological content gaps with reference to tools discussed earlier within the accounting curriculum offered by higher learning institutions. Additional research questions that this paper aimed to address were as follows:

• What is the level of awareness of classroom teaching technologies among accounting educators?
• What are the antecedents for technology inclusion into existing Institutions of Higher Learning (IHL) curriculums?
• What are the problems associated with technology implementation?

## Methods

This was an exploratory study with the main focus of understanding how much technological context was covered in the existing accounting curriculum. Throughout the study, Table 2 (detailed description of digital tools) was used as a reference for experts to evaluate and benchmark best practices. A total of 80 individuals, comprising of experts, academicians, program counselors, curriculum experts, program coordinators, subject experts, industry advisory panel members and practitioners were interviewed (with their consent) for feedback. Detailed curriculum and program structures were also reviewed for comparison. The authors Saravanan Muthaiyah and Sanjaya Chamara Sembakutti conducted the interviews; only participants who were willing took part were interviewed, the rest left the hall. The interview took about 10 minutes for each person. We prompted the respondents to talk about the level of digital awareness among instructors and express freely on what were challenges that they faced in updating existing curriculum. We took about 5 days to collect the information needed. Notes were taken during the sessions.

**Table 2.** Description of classroom technologies.

Classroom technologies for future accountants	Brief description
SAP (ERP- enterprise resource planning)	SAP FICO (FI – SAP Finance and CO – SAP Control) is a core module in the SAP ERP Central Component. It describes cost elements, cost centers, profit centers (handles cost data related to line of business), profitability analysis, product costing and internal orders.
IBM Cloud	IBM provides educators with software and courseware using its cloud infrastructure. This includes data gathering, sharing, computation, processing and simulation. Students can experiment with what-if analysis and virtual simulations.
Bloomberg	Bloomberg (supported by Classroom Inc.) is a non-profit online educational service. Bloomberg includes simulations and technology tools for banking, economics, finance and accounting.
Microsoft Dynamics 365	Microsoft Dynamics is a combination of ERP and CRM.
XBRL (Extensible Business Reporting Language)	XBRL is based on XML (Extensible Markup Language) which is a standard protocol for Internet communication. It is designed to allow financial and non-financial data to be exchanged for business reporting.
Idaciti	Idaciti makes global financial data usable, accessible and shareable. It can also be used for XBRL financial datasets.
Aura and e-audit	Aura and e-audit specifically support competencies for the area of audit in the digital age.
DMS – document management system	In the area of taxation competencies for the digital age, DMS is highly recommended.
Accounting information systems (AIS)	The AIS module includes six major components: personnel, hardware, software, procedure, stored data, procedures and internal controls.
Cyber security and trusted computing	One very key area that institutions must prepare their students for is the area of trusted computing and cyber security.
Cryptocurrencies and electronic payment systems (CEPS)	Another area that has heavily impacted movement of funds and cash flow is electronic payment methods.

### Ethical approval

Ethical approval was obtained from the research management center at the university. Researchers had to first submit the title of the project, what the author planned to do for the interviews and details of study objectives. The officer at the research management center after reviewing the documents will then issue a letter of clearance for the data collection to be carried out. The approval letter was then obtained, and the reference number of this letter is EA1212021. Consent was obtained verbally prior to the interview and only respondents who were agreeable to be interviewed were approached. The reason for this was this was done in conjunction to a digital transformation workshop in which academics were allowed to express themselves freely about issues and challenges with regards to the teaching curriculum. The review board approved this as it was on voluntary basis.

## Results

### Awareness of emerging technologies for teaching

Table 3 below shows how much instructors were aware of tools that could be used to teaching emerging arrears. For this section we interviewed 52 people inclusive of subject matter experts and instructors. We selected those who had at least 5 years of teaching experience. In total, 58 % of educators were not aware about most of the technologies discussed earlier. ^11^ Among those who said they were aware, 23% had only superficial knowledge. For instance, some instructors did not know that Bloomberg Lab provided datasets that could be used for big data and predictive analytics.

**Table 3.** Technology awareness among instructors.

Level of awareness	Working experience	Frequency	Cumulative percent
**Aware**	5-6 years	6	**23%**
	5-7 years	4	
	> 8 years	2	
**Not aware**	5-6 years	11	**77%**
	5-7 years	14	
	> 8 years	15	
	**Total**	**52**	

### Antecedents of implementing classroom technologies

Interviewees were requested to rank order factors for the implementation of emerging teaching technologies in the accounting curriculum (see Table 4). Specific reasons that were listed included: 1) training on specific technologies; 2) cost for software licensing; 3) technological resources available (computer labs and hardware); 4) compliance on ministry and accounting body standards; 5) program sustainability; and 6) others.

**Table 4.** Antecedents for classroom technology implementation.

Criteria	Factors	Rank *r*	Aggregate *n*	Percentage %
**Most significant**	training on specific technologies	6	108	22.5
**2** ^**nd**^ **most significant**	cost for software licensing	5	85	21.3
**3** ^**rd**^ **most significant**	technological resources available	4	60	18.8
**4** ^**th**^ **most significant**	ministry and accounting body standards compliance	3	39	16.3
**5** ^**th**^ **most significant**	program sustainability	2	10	6.3
**Least important**	Others	1	2	2.5
			**80**	**100**

The most significant factor was assigned six points, followed by the second most significant value with five points and so on. The results show the overall ranking of the reasons that contributed to the antecedents of classroom technology implementation. Data shows that training on specific technologies was the most significant contributor.

### Challenges related to implementation of classroom technologies

Table 5 highlights the overall ranking of further issues faced by IHL with regards to the inclusion of classroom technologies into the curriculum. The most important factor was assigned four points, followed by three points for the second most important factor and so on.

**Table 5.** Challenges for technology implementation.

Performance evaluation	Challenges	Rank *r*	Aggregate *n*	Percentage %
**Most important**	Cost to the university	4	100	31.3
**2** ^**nd**^ **most important**	Retraining staff to be competent	3	75	31.3
**3** ^**rd**^ **most important**	Realignment of course structure	2	40	25
**4** ^**th**^ **most important**	Management and peer support	1	10	12.5
			**80**	**100**

### Hypothesis testing

Table 6 summarizes key hypotheses that were formulated to facilitate this study. In this hypothesis, the objective is to find out whether the implementation of digital content is desirable, the impact of regulatory requirements and will it improve job possibilities significantly.

**Table 6.** Hypotheses.

**Hypothesis 1**	Digital content is significant for the accounting curriculum
**Hypothesis 2**	Regulatory requirements and program standards have a significant relationship with the inclusion of technological content
**Hypothesis 3**	Technological competency is highly desirable among potential employers

**Table 7.** Response to necessity of digital content.

		Frequency	Percentage
**Valid**	Yes	62	78%
	No	18	22%

**Table 8.** Response to digital content yielding benefits.

		Frequency	Percentage
**Valid**	Yes	72	90%
	No	8	10%

**Table 9.** Response to digital content as standard practice.

		Frequency	Percentage
**Valid**	Yes	60	75%
	No	20	25%

**Table 10.** Regulatory requirements have significant relationship with digital inclusion.

		Frequency	Percentage
**Valid**	Yes	71	89%
	No	8	11%

**Table 11.** Competency desired by employers.

		Frequency	Percentage
**Valid**	Yes	74	92.5%
	No	6	7.5%

The questions that were developed to support the hypothesis are:

*Hypothesis 1 - digital content is significant for the accounting curriculum*

• Do you think digital content inclusion is necessary?
• Will digital content inclusion yield benefits to accounting graduates?
• Digital content inclusion should be standard practice.

Overall, 78% of the experts agree that digital content is necessary, 90% of the respondents agree that digital content will yield benefits to accounting graduates, and lastly, 75% of the respondents agree that this should be standard practice. In this context, the hypothesis that digital content is significant for improved accounting curriculum can be accepted.

*Hypothesis 2 – regulatory requirements and program standards have a significant relationship with the inclusion of technological content*

Out of 80 experts interviewed, 89% agree that regulatory requirements have a significant relationship with the inclusion of technology content. Therefore, this hypothesis can be accepted as well.

*Hypothesis 3 –Technological competency is highly desirable among potential employers*

Out of 80 respondents, 92.5% agree that competency requirement among potential employers has a significant relationship with the inclusion of technology content in the curriculum. Therefore, this hypothesis can be accepted as well.

## Conclusions

Results highlight that there is a significant mismatch of what is needed with what is being taught at universities today. Our hypothesis on technology competency, program standards and digital content required supports this as well. Insights derived from the mapping of existing syllabi enabled us to understand the lack of digital inclusion described earlier in the teaching pedagogy. In summary the findings helped us to provide suggestions as to how universities can improve existing curriculum offered to students. This included eleven essential areas of know-how can be fairly distributed across subjects from year one to year four for a four-year degree program. We are confident that changes made to the program structure and curriculum will definitely produce future ready graduates.

## Data availability

Figshare: FinTech What Should be Taught Really? https://doi.org/10.6084/m9.figshare.14871168.v1. ^11^

This project contains the following underlying data:

• DataSet FINTECH.xlsx (Dataset includes responses that were documented during the Interview of panel experts, academicians, program counselors, curriculum experts, program coordinators, subject experts, industry advisory panel and practitioners. A total of 15 institutions have been listed and categorically labelled as antecedents, challenges and hypothesis.)

Data are available under the terms of the Creative Commons Zero “No rights reserved” data waiver (CC0 1.0 Public domain dedication).
