# Supplementary material for: Bridging skill gaps and creating future ready accounting and finance graduates: an exploratory study
Source: F1000Res. 2021 Sep 6;10:892. [Version 1] doi: 10.12688/f1000research.72880.1 (PMC8753577; doi:10.12688/f1000research.72880.1)
Supplement: Peer review history

# Peer review: Reviewer response for version 1

**DOI:** 10.5256/f1000research.76488.r98790

**Published:** 2022-01-11

**Authors:** Samuel Jebaraj Benjamin

**Affiliations:** 1 School of Accounting, Finance and Economics, University of Waikato, Hamilton, New Zealand

Comments;

 This is an interesting study and timely. Fintech and/or digital skills are highly important for accounting and finance graduates, but is still lacking in their undergraduate syllabus. This study has the potential to contribute to our understanding of this issue. The following are the main issues that the paper needs to address in order to ensure its rigour and contribution are noteworthy. 

1. You do not define the scope of digital transformation or fintech. Technological areas such as AI, blockchain, cloud computing, ANN, etc. are thrown into the mix without clear coherence with the key focus of the paper. It is hard to follow through chronologically the flow of arguments and paragraph 1 of the Literature Review seems to have been written haphazardly. I suggest to more clearly outline the arguments and motivations of the paper with the proper citations and focus.
2. “Today’s accounting curriculum is focused on descriptive accounting (see Figure 1). Prescriptive accounting is rather limited, with the exception of managerial and cost accounting only (see Figure 2). For example, assets are still recorded and maintained at cost which is not aligned to fair value accounting principles” ;
  The relevancy of the statement above is unclear. You are talking about an accounting treatment issue and this has got nothing to do with the fintech or digitalization that your study is concerned with. 
3. I do not see the relevancy of Figure 1 and 2 towards the aims of this study.
4. It is unclear why many of the items mentioned in Table 1 were identified, as no prior discussion about it is made in the preceding sections. Only concepts, such the ones I mentioned in Point 1 above, are discussed, but, in Table 1, most of the areas are entirely new in the paper and not previously defined nor addressed. Table 2 should appear before Table 1. However, it is still unclear if how the ‘technologies’ mentioned in Tables 1 and 2 fit under the grand idea of ‘fintech’ or some of the sub-areas that you mentioned in paragraph 1 of the Literature Review.
5. Hypotheses testing approach and how the conclusions are arrived (i.e., valid or not valid) for each hypotheses have to explained. 
6. Is the scope of your study confined to only the undergraduate syllabus in accounting and finance or include postgraduate? You need to clearly indicate this.

Is the work clearly and accurately presented and does it cite the current literature?

Partly

If applicable, is the statistical analysis and its interpretation appropriate?

Partly

Are all the source data underlying the results available to ensure full reproducibility?

Yes

Is the study design appropriate and is the work technically sound?

Yes

Are the conclusions drawn adequately supported by the results?

Yes

Are sufficient details of methods and analysis provided to allow replication by others?

Partly

Reviewer Expertise:

Accounting, finance

I confirm that I have read this submission and believe that I have an appropriate level of expertise to confirm that it is of an acceptable scientific standard.

# Peer review: Reviewer response for version 1

**DOI:** 10.5256/f1000research.76488.r93576

**Published:** 2021-12-23

**Authors:** Indrawati Sambas

**Affiliations:** 1 Faculty Economics & Business, Telkom University, Bandung, Indonesia

I think this paper is good and is an imperative addition to the literature. I found that the work was clearly and accurately presented, and it cites the current literature. The study design was appropriate and the work is technically accepted. The work should elaborate more on the explanation of Figure 1 and 2, to make it clearer. The writers have to expand on how the hypotheses are accepted or rejected. What is the threshold? Please cite business research books such as Sekaran (2016). It is better if the verbatim interview can be accessed, to make the explanation of the findings clearer.

Is the work clearly and accurately presented and does it cite the current literature?

Yes

If applicable, is the statistical analysis and its interpretation appropriate?

Yes

Are all the source data underlying the results available to ensure full reproducibility?

Yes

Is the study design appropriate and is the work technically sound?

Partly

Are the conclusions drawn adequately supported by the results?

Yes

Are sufficient details of methods and analysis provided to allow replication by others?

Yes

Reviewer Expertise:

Customer adoption in services based on technology

I confirm that I have read this submission and believe that I have an appropriate level of expertise to confirm that it is of an acceptable scientific standard.

## References

[1] : Research Methods For Business. *Wiley* .2016;
